# Acute monoarthritis in a delayed diagnosis of syphilis patient with persistent rupioid psoriasis-like lesions

**DOI:** 10.1186/1471-2334-12-338

**Published:** 2012-12-06

**Authors:** Kejian Zhu, Qiang Zhou, Rui Han, Hao Cheng

**Affiliations:** 1 Department of Dermatology, Sir Run Run Shaw Hospital, Zhejiang University School of Medicine, No. 3 Qingchun Road East, Hangzhou, Zhejiang, People’s Republic of China

**Keywords:** Syphilis, Monoarthritis, Rupioid psoriasis-like plaque

## Abstract

**Background:**

The incidence of syphilis is increasing in many parts of the world. Clinicians may have limited experience in the diagnosis when the clinical appearance is unusual. If early diagnosis is not made and prompt treatment not given, then the disease may remain quiescent until more serious symptoms or systemic involvement develops.

**Case presentation:**

We report the first case of a delayed diagnosis of syphilis with a ten-year history of persistent rupioid psoriasis-like lesions. Acute monoarthritis and high fever together with aggravation of skin lesions led to a careful clinical examination. Skin biopsies demonstrated syphilis spirochetes on immunohistochemical stain, and syphilis serological titers were positive. Standard treatment with benzathine penicillin brought a partial and transient improvement. A complete clinical and serological resolution of the disease was achieved by a prolonged and repeated penicillin treatment combined with methylprednisolone. A 7-year follow-up of the patient proved a full recovery.

**Conclusion:**

Our case highlights the fact that clinical signs of syphilis can be diverse and complicated. Unusual clinical manifestations can happen in an immunocompetent individual. Treatment strategy may need to be adjusted in a difficult case.

## Background

Syphilis is an infectious sexually transmitted disease, caused by *Treponema pallidum*. It continues to be a public health problem, with an estimated 12 million new cases per year worldwide [1]. In many developing countries, as much as 10% of the population may be infected [2]. The clinical manifestation of syphilis varies in appearance. In addition to common muco-cutaneous characterizations, some atypical or uncommon presentations mimicking pseudolymphoma [3], pemphigus vulgaris [4], erythema multiforme [5], cutaneous vasculitis, and Reiter’s syndrome [6] have been reported. We here report a unique case of syphilis presenting with persistent rupioid psoriasis-like plaques and acute monoarthritis of knee joint.

### Case presentation

A 69-year-old man presented to our hospital in August 2004 for an evaluation due to one month history of right knee joint pain with fever, and two plaques showing on his abdomen and scalp for 10 and 2 years, respectively. The lesion on the abdomen began as an asymptomatic small light red macule following scratch. It enlarged gradually over the past ten years, and formed an unhealed ulceration plaque with thick crusts on its surface. He was seen by several different dermatologists in the past, but no definitive diagnosis was given. He had a failed trial of topical antibiotics. Two years earlier, an additional similar lesion on his front scalp developed from a linear wound due to hair shaving. One month prior to his visit, he developed an acute onset of right knee joint pain and swelling with decreased range of motion that was worsening with activity. Ten days after the onset of joint pain, he spiked a fever of up to 38.7°C, and went to a local hospital where he was diagnosed with acute arthritis and skin ulcers. He was treated with clindamycin and indomethasone and discontinued them after two weeks of no response. He also had 5 kilograms of unintentional weight loss in one month. The patient’s past medical history includes chronic bronchitis, 20-year history of Parkinson’s disease, and 30-year history of ankylosing spondylitis. He had no painless ulcer or rash on his penis and scrotum, urethral discharge, blurred vision or headache. He admitted engaging in several acts of unprotected heterosexual intercourse, with the last sexual encounter occurring 3 years prior. He had never traveled out of the province.

**Figure 1:**
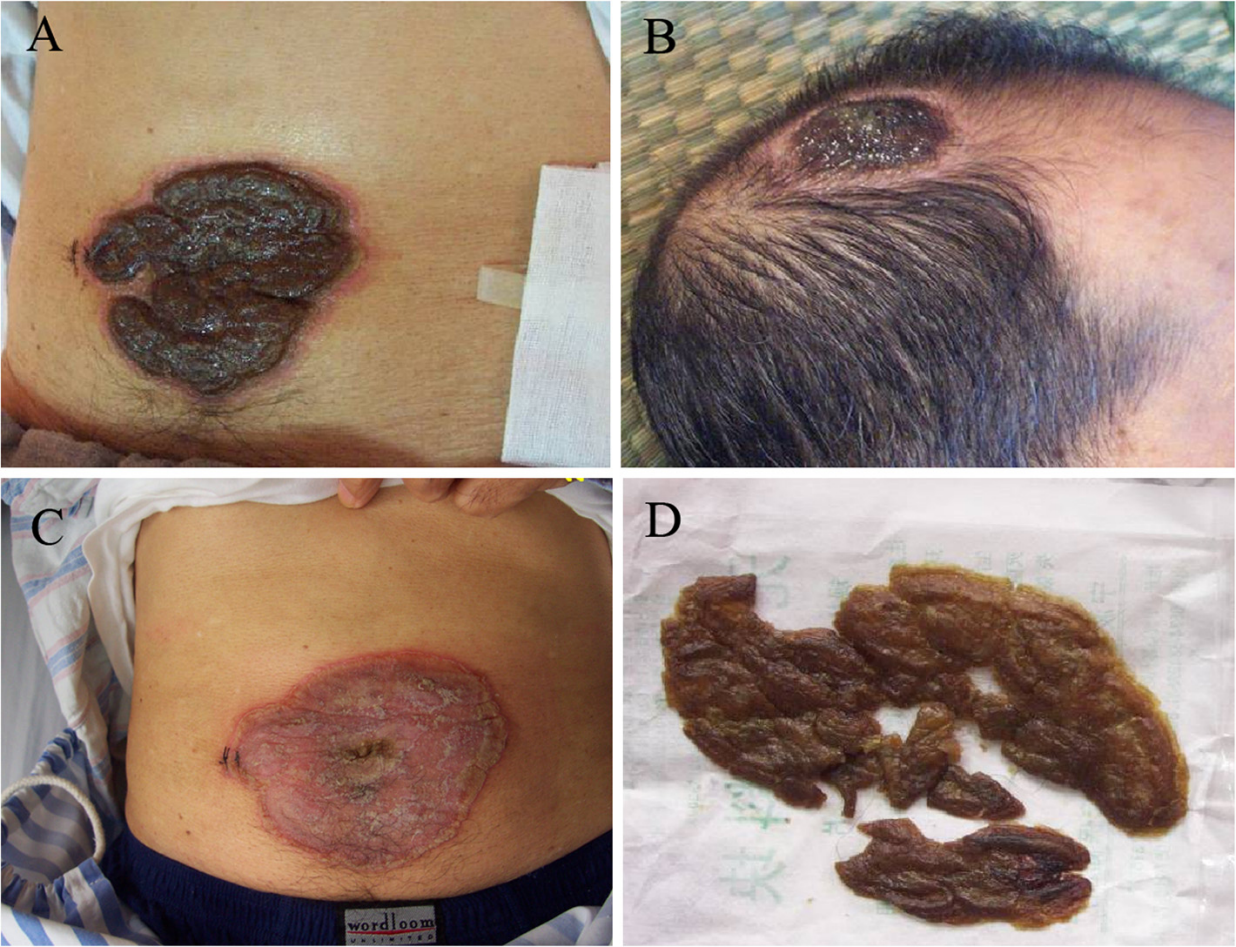
**Rupioid lesions of erythematous plaques covered with thick and tightly-adherent dirty-appearing crusts on the abdominal wall for 10 years (panel A) and on the frontal aspect of the scalp for 2 years (panel B).** Well demarcated red, rough and scaling surface of the plaque on the abdominal wall after completion of 3 doses of benzathine penicillin (panel **C**) and the shed crusts (panel **D**).

**Figure 2:**
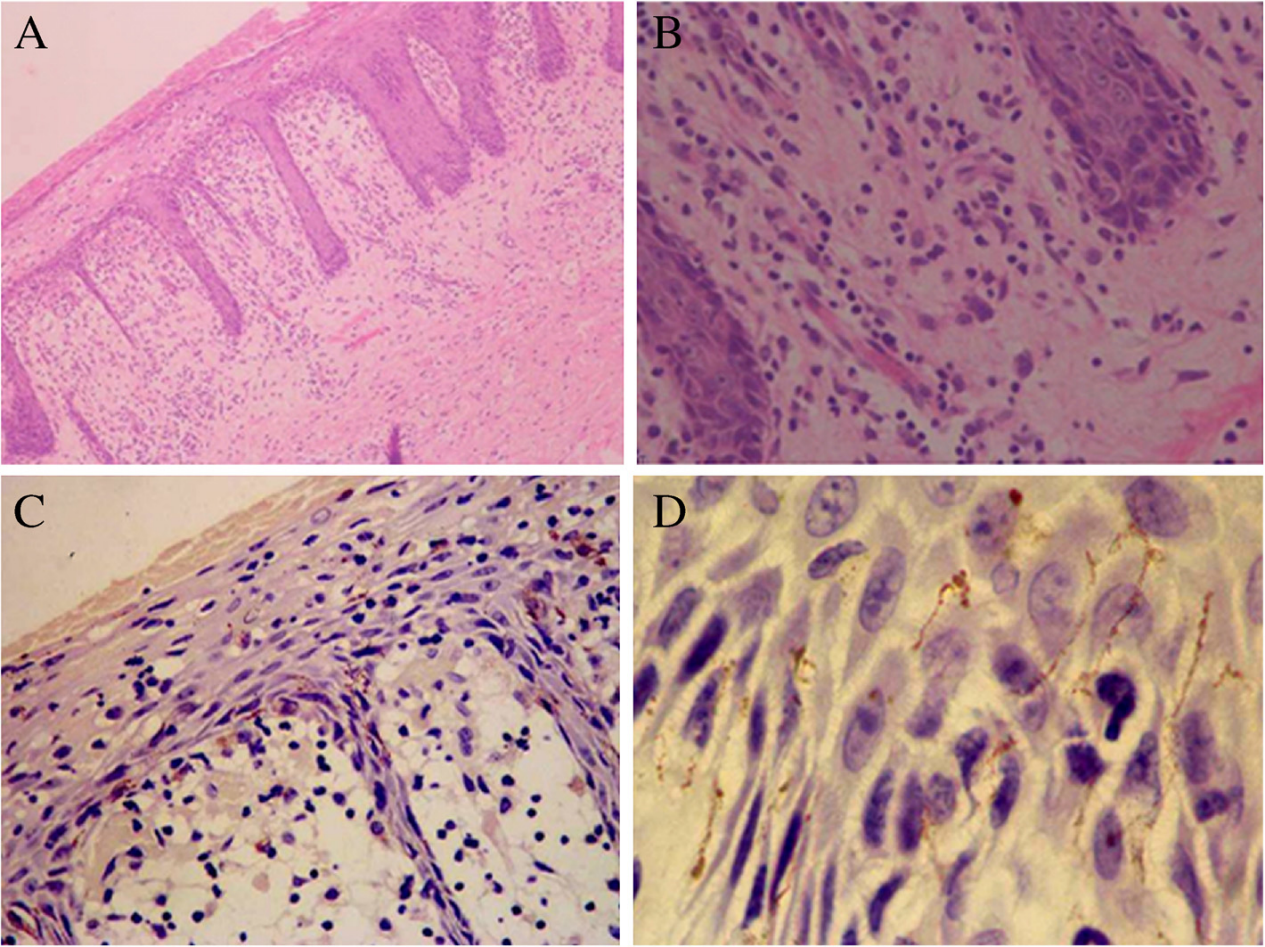
**Psoriasiform pattern of inflammation and perivascular infiltration containing lymphocytes, monocytes and numerous plasma cells**. Haematoxylin and eosin stain: original magnification (panel **A**)×100 and (panel **B**) ×400. Spiral and thread-like organisms highlighted by brown chromogen representing spirochetes were observed within the lower layers of the epidermis, and dermo-epidermal junction and upper dermis. Immunohistological stain: original magnification (panel **C**)×100 and (panel **D**) ×400.

Physical examination on admission revealed a fever of 38.5°C. Two solitary erythematous plaques were observed with rupioid aspect covered with thick, tightly-adherent, dirty-appearing crusts, including one on his abdomen measured about 10×10 cm^2^ with irregular, boggy and necrotic border (Figure 1A), and the other one on his front scalp measured as 3×4 cm^2^ in size (Figure 1B). Right knee exam revealed swelling, warmth, redness and tenderness. Laboratory analysis showed a C-reactive protein (CRP) of 9mg/dL and erythrocyte sedimentation rate (ESR) was >140mm/hr. HLA-B27 test was positive. Cell counts, urine analysis, hepatic panel, fasting blood glucose, serum immunoglobulin, tumor markers of carcinoembryonic antigen (CEA), alpha-fetoprotein (AFP), cancer antigen (CA)-199, and prostate specific antigen (PSA) were within normal limits. Serum rheumatoid factor, anti-nuclear antibodies, anti-neutrophil cytoplasmic autoantibodies, anti-tuberculosis antibody and PPD test, and blood culture were negative. Further studies revealed negative HIV antibody by enzyme-linked immunosorbent assay, positive rapid plasma reagin test (RPR) titer 1:16, and positive reactive treponema pallidum hemagglutination assay. CD_3_ proportion was 70.8% (normal range 60-85%), CD_4_ 52.3% (normal range 24.5-48.8%), CD_8_ 22.8% (18.5-42.1%) and the CD_4_/CD_8_ ratio was 2.3 (normal range 1.5-2.1). Urethral swab culture was negative for gonorrhea, chlamydia and mycoplasma. Right knee ultrasound revealed effusion of the posterior patellar tendon, and MRI scan showed lateral and medial meniscus degeneration, suprapatellar bursa and joint space effusion. Microscopic study of thick, turbid and yellowish synovial fluid aspirated from his right knee showed white blood cells >200/HP, red blood cells 5-10/HP, but no crystals or organisms. Culture of joint aspirated specimen collected before antibiotics revealed 100% staphylococcus epidermidis with sensitivity to clindamycin, rifampin and vancomycin. Abdominal rash biopsy showed irregular psoriasiform epidermal hyperplasia with overlying crust and confluent parakeratosis containing many neutrophils, degenerative and necrotic keratinocytes, papillary dermis edema with dense lymphohistiocytic infiltration containing numerous plasma cells, scattered eosinophils and neutrophils without granulomatous process (Figure 2A and B). Immunohistochemical studies showed no evidences of lymphoma. Culture of the skin biopsy was negative for bacteria, mycobacterium tuberculosis and fungi. Silver staining for treponema was not conducted due to technical difficulties. Syphilis and septic arthritis were diagnosed based on clinical features, histological and laboratory findings.

Cefmetazole was administered intravenously on the second day of admission after collecting blood, aspirated and skin biopsy specimen. However, he continued having fever. His knee joint symptoms and skin rashes remained unchanged after 5-day of treatment. Infection disease service was consulted on day-6 of admission. He was recommended to receive intravenous norvancomycin for 5 days, but that showed no improvement. On day-12 of hospitalization, intramuscular benzathine penicillin 2.4MU was administered in a form of 3 doses with 1 week apart after getting a diagnosis of syphilis. One week after intramuscular benzathine penicillin he returned to afebrile, and the severity of his knee pain and swelling reduced significantly. Additionally, the thick crusts covering two plaques gradually shed and finally left well-demarcated red, rough and scaling surface (Figure 1C and D). His skin lesions and joint pain continued improving and he was discharged after completing a course of penicillin. Repeated labs showed CRP 2.4mg/dL, ESR 32mm/h, and RPR1:8. His skin lesions continued improving with some desquamation and postinflammatory hyperpigmentation, and his knee problem was completely resolved over the following one month.

Two months after discharge, the patient was readmitted due to a fever of 39.2°C, recurrent right knee pain and swelling with the flare-up skin lesions. Repeated laboratory tests showed marked inflammation process (CRP, 7.6mg/dL and ESR, >140mm/h). Blood cell counts were within normal limits. Blood and skin rash specimen revealed no organisms. Urethral swab culture for gonorrhea, chlamydia and mycoplasma were negative. RPR was 1:8, and HIV was negative. Right knee MRI scan showed subcutaneous edema with no joint effusion. Symptoms were improved after a consecutive 10-day intravenous benzylpenicillin. The patient left hospital against medical advice, and did not complete the course of antibiotics. One month later, he was sent back to hospital by his family with a fever of 39.6°C, suddenly enlarged skin lesions, and a newly developed groin dark red plaque (2×3 cm in size) with thick crusts. Right knee pain and edema significantly limited his gait and range of motion. He took ibuprofen with minimal improvement. He had no leukocytosis, labs showed CRP 15.5mg/dL, ESR 126mm/h, RPR 1:16, and negative HIV antibody. Right knee MRI scan revealed a similar finding as found at his first admission. Culture of the knee aspirate revealed no growth of bacteria. A skin biopsy from the newly formed groin lesion showed features compatible with the finding of his abdominal skin biopsy. Immunohistochemistry (IHC) stain by using a rabbit/mouse polyclonal anti-T. *pallidum* (1:200, Biocare Medical, CA, USA) demonstrated the presence of syphilis spirochetes in both skin lesions (Figure 2C and D). Typical spiral and thread-like organisms highlighted by brown chromogen were observed in the lower layers of the epidermis, at dermo-epidermal junction and upper dermis with a perivascular pattern. Due to elevated RPR titer of 1:8 to 1:16 and syphilis spirochetes found in the skin biopsy and joint aspirate, intramuscular benzathine penicillin 2.4MU was given once a week for consecutive 4 weeks and doxycycline 100mg twice daily for 28 days. Over the next few days, the pain was increasing leading to difficult sleeping. Methylprednisolone 16mg daily was added to the therapeutic regimen. His fever, skin lesions and joint pain were gradually improved in 2 weeks. He was weaned off methylprednisolone over a period of 6 weeks after his symptoms all gradually resolved. A repeated RPR test performed 3 months after treatment was 1:4. Two years later, RPR became negative. Follow up visits of the patient continued for the next 7 years. No relapse of the symptoms occurred, indicating the patient’s successful treatment and full recovery.

## Conclusions

As a great masquerader, cutaneous lesions of secondary syphilis are highly variable. The cutaneous manifestations that characterize secondary syphilis are usually superficial and include four major types of rashes: macular, papular, papulosquamous, and pustular [4]. Hyperkeratotic, crusted limpet like and discolored lesions called rupia are rare and have been seen in relapsing secondary syphilis [7] and in several other situations, most of which involve varied degrees of immunesuppression, including HIV, malnutrition, pregnancy, or diabetes [8]. In the present report, we described a case with an appearance similar to that of a rupioid psoriatic skin lesion and associated knee joint symptoms occurring in an immunocompetent elderly male patient with serology confirmed syphilis and immunohistochemistry proved positive spirochetes in his skin lesions.

Given the presence of plaques with sharp borders, centrally covered dark rupioid crusts on the abdomen, scalp and groin in our patient and especially the sudden onset of fever, accompanying arthritis and weight loss, malignant syphilis was an entity that should be considered. However, our patient did not have any preceding papulopustular change and ulcer formation mostly seen on the face and limbs, no rapid evolution of disseminated skin lesions were observed. Additionally, the patient’s systemic symptoms progressed many years after the skin involvement rather than a prodrome of the skin manifestation, no medium-vessel vasculitis found in skin biopsy. There was also no evidence for HIV infection or diabetes. Therefore, a diagnosis of malignant syphilis is less likely. Secondary syphilis occurs weeks to months after exposure to infection and tertiary syphilis can follow the initial infection by 3 to 15 years. Although the present case had an impressive persistent big crusted plaque for ten years which is much longer than the natural history of secondary syphilis, the histological feature was fully consistent with the change of secondary syphilis [9] and was lacking granulomatous inflammation with palisaded macrophages and fibroblasts which typically would be seen in tertiary syphilis [10]. Spirochetes were found by immunohistochemistry in our patient’s skin lesions whereas they are rarely visualized in patients with tertiary syphilis [11]. Thus, we consider that the diagnosis of secondary syphilis would be more reasonable than tertiary syphilis

Diagnosing syphilis in HIV-positive patients remains a clinical challenge since syphilis in such patients frequently manifests with atypical features and chronology [12]. Our HIV-negative patient had a longstanding and progressing 10-year’s history of rupioid skin lesions and orthopaediac involvement but absence of primary syphilis, renders diagnostic difficulty. This case is noteworthy in that, to our knowledge, it is the first report of syphilis in a HIV-negative patient in whom the skin manifestation of secondary syphilis closely resembles that of rupioid psoriasis and its aggravation correlates well with joint symptoms.

This patient was admitted to hospital in three different occasions presenting with fever and painful swollen right knee joint, suggesting an acute monoarthritis. This is most likely caused by infection, trauma, or crystal disease. The patient has no history of traumatic injury, and no crystals were found in synovial fluid. A diagnosis of septic arthritis was suspected due to positive staphylococcus epidermidis from knee aspirate culture and the lab findings of elevating inflammation markers on his first admission. But neither the initial cefmetazole nor the targeted antimicrobial agent norvancomycin chosen according to the antibiotics sensitivity test improved the joint involvement. Whereas benzathine penicillin dramatically resolved not only the skin lesions but also the joint pain and swelling after a diagnosis of syphilis was given based on serum RPR titer and positive TPPA along with cutaneous histopathology features. Polyarthritis with synovitis associated with early syphilis has been described and usually occurs 3–12 weeks into the secondary stage [13]. But our patient’s manifestation was atypical because his monoarthritis occurred 10 years after the appearance of the first syphilitic skin lesion. Recently, a case of acute mono-arthritis secondary to syphilis was reported [14]. Although monoarthritis is rare in secondary syphilis, our case highlights the fact that systemic involvement including fever, acute arthritis and weight loss may occur after a long quiescent phase if syphilis is undiagnosed and remains untreated.

The secondary syphilis represents hematogenous dissemination of *T. Pallidum*[15]. All three crusted plaque lesions in our patient presented on the site of injury. In Fuehrer’s report [16], the lesion of their patient was a suppurative plaque on an apparent insect bite several months before. This suggests that secondary syphilis skin lesions could be triggered by trauma and manifest as a Koebner response. Thus, mucocutaneous lesions in secondary syphilis might be a reaction of specific hypersensitivity to the treponemal infection seems to be reasonable. However, we would suspect that it might be caused by direct *T. pallidum* invasion rather than by an allergic reaction based on our findings of typical spiral and thread-like organisms by immunohistochemistry in both a very old and a newly developed lesion in our present case. And also, the two lesions developed in the recent years may have resulted from the inoculation of spirochetes in the first lesion that existed for ten years since the patient’s admitted last sexual encounter occurred 3 years prior. This is supportive for the diagnosis of secondary syphilis because tertiary syphilis is not infectious.

The acute arthropathy of the right knee joint was initially thought to be septic arthritis due to reported 100% stahphylococcus epidermidis from synovial fluid culture, but adequate treatment with anecdotal cefmetazole and following norvancomycin was proved to be unresponsive. Since staphylococcus epidermidis is a common human skin flora and there is no evidence of immunocompromise in our patient by repeated extensive lab workup, we deduced a false positive synovial fluid culture which misled treatment as for joint bacterial infection. After giving syphilis standard therapeutic regimen with benzathine penicillin, his knee symptoms together with the skin lesions resolved completely. Therefore, syphilitic synovitis was given as a working diagnosis. But his two episodes of arthropathy relapse shortly after discontinued penicillin treatment along with elevated inflammatory markers of CRP and ESR, as well as the fact that the arthropathy was healed with 7-year follow-up by repeated procedures of benzathine penicillin and 6-week methylprednisolone prompted us to consider the joint symptoms to be reactive rather than septic. Although it is well known that both gonococcal and nongonococcal infections may lead to aseptic “reactive” arthritis or Reiter’s syndrome [17], reactive arthritis due to syphilitic infection is quite rare. It has been reported that the inheritance of HLA-B27 confers a relative risk of 30–50 times for development of sexually transmitted arthritis [17]. Our patient has a past history of ankylosing spondylitis and his HLA-B27 was proved to be positive. This212121 may be responsible for the provocation of syphilis reactive arthritis.

According to the 2006 American CDC guidelines [18] and the 2001 European guidelines [19], penicillin is still the treatment of choice for all stages of syphilis. We administered intramuscular benzathine penicillin 7.2MU, as three doses for 2.4MU each with a week apart, but the recurrence of skin lesions and new skin rash development with rise of nontreponemal titers were indicative of treatment failure. Considering the possible resistance to penicillin, we added oral doxycycline in addition to the regimen of repeating penicillin injection. This finally led to a successfully clinical and serological recovery. It has been postulated that treatment for late latent syphilis and tertiary syphilis might theoretically require a longer duration of therapy because organisms are dividing more slowly [18]. The experience of our patient seems to imply that secondary syphilis with longstanding cutaneous eruptions for many years and especially while accompanied by systemic presentations such as fever and arthralgia could be difficult to treat and prolonged or combined antibiotics may be needed.

The current case provides many interesting and important points. Unusual rupioid psoriatic lesions may present in an immunocompetent individual with no evolvement for many years. Injury could be the trigger for the development of this special type of secondary syphilitic cutaneous manifestation. Systemic features, especially the acute onset of monoarthritis with a reactive entity may occur in an untreated syphilis patient ten years after skin involvement. Standard treatment regimen seems to be not effective. Prolonged and repeated treatment with penicillin as well as combined antibiotics may be needed to achieve a complete clinical and serological resolution of the disease. We should be aware that the clinical signs of syphilis can be diverse and complicated. If it can be recognized early we then can treat it promptly.

### Consent

Written informed consent was obtained from the patient for publication of this Case report and any accompanying images. A copy of the written consent is available for review by the Series Editor of this journal.

## Competing interests

The authors declare that they have no competing interests.

## Authors’ contributions

KZ took care of the patient and drafted and revised the manuscript. QZ and RH have been involved in patient clinical care and the interpretation of data. HC took care of the patient and reviewed the manuscript. All authors read and approved the final manuscript.

## Pre-publication history

The pre-publication history for this paper can be accessed here:

http://www.biomedcentral.com/1471-2334/12/338/prepub
